# Regulative Mechanism of Guanidinoacetic Acid on Skeletal Muscle Development and Its Application Prospects in Animal Husbandry: A Review

**DOI:** 10.3389/fnut.2021.714567

**Published:** 2021-08-12

**Authors:** Zhaoming Yan, Zhaoyue Yan, Shuangli Liu, Yunju Yin, Tai Yang, Qinghua Chen

**Affiliations:** ^1^College of Animal Science and Technology, Hunan Agricultural University, Changsha, China; ^2^Chemistry Department, University of Liverpool, Liverpool, United Kingdom

**Keywords:** guanidinoacetic acid, creatine, muscle development, gene regulate, livestock and poultry

## Abstract

Guanidinoacetic acid is the direct precursor of creatine and its phosphorylated derivative phosphocreatine in the body. It is a safe nutritional supplement that can be used to promote muscle growth and development. Improving the growth performance of livestock and poultry and meat quality is the eternal goal of the animal husbandry, and it is also the common demand of today's society and consumers. A large number of experimental studies have shown that guanidinoacetic acid could improve the growth performance of animals, promote muscle development and improve the health of animals. However, the mechanism of how it affects muscle development needs to be further elucidated. This article discusses the physical and chemical properties of guanidinoacetic acid and its synthesis pathway, explores its mechanism of how it promotes muscle development and growth, and also classifies and summarizes the impact of its application in animal husbandry, providing a scientific basis for this application. In addition, this article also proposes future directions for the development of this substance.

## Introduction

The body composition of mammals includes skin, muscle, fat and bones, among which the proportion of skeletal muscle is more than 40%, making it the largest organ in the body. In addition to maintaining exercise capacity, body balance and respiratory function, skeletal muscle also acts as an endocrine organ to secrete a variety of cytokines to mediate the interaction between cells and perform diverse biological functions (1, 2).

The basic functional unit of skeletal muscle is myofiber, and its development is closely related to the identification and differentiation of myoblasts (3). The amount of protein consisted in myofibers is about 50–75% of the total amount of protein in the animal body. Under normal physiological conditions, the protein synthesis and degradation rates of myofibers are in a relatively balanced state. When the state is out of balance, the efficiency of protein synthesis is higher than degradation, an overall outcome of increased muscle mass will be shown. No matter humans or animals, there are many factors that may disturb the equilibrium of the state, including nutrition regulation, exogenous nutrient intervention, and regular exercise. The method of exogenous nutrient intervention has gradually grabbed increasingly attention in this field (4).

It is well-known that guanidinoacetic acid (GAA) could be used as a nutritional supplement to promote muscle development and increase the body's energy reserves. Human dietary supplementation with GAA helps to increase muscle strength and endurance, and enhance athletic performance and release fatigue (5). Currently, creatine (Cr) and its precursor GAA have also been used in medical research for the treatment of muscle atrophy and neurodegenerative diseases (6). Furthermore, with the continuous improvement of people's living standards, the dietary structure has undergone significant changes, and the demand for meat product shifted from being quantity-satisfying to quality-ensuring. Therefore, improving the quality of meat products is an important task of today's animal husbandry (7, 8). The addition of GAA in animal diets helps to delay the rate of glycolysis on the basis of increasing muscle production so that the meat quality could be improved (9, 10). However, at present, the mechanism of GAA in promoting myofiber development and growth has not been fully elucidated. This article reviews the physical and chemical properties, mode of action, application effects and prospective development of GAA. It is expected to provide a theoretical basis for the application of GAA in human health and animal husbandry production.

## Properties of Guanidinoacetic Acid

GAA is an immediate precursor substance of Cr and its phosphorylated derivative (phosphocreatine, Cr-P) synthesized in the body of animals (11). It was first isolated from the urine of humans and dogs. Cr accepts pyrophosphate from adenosine triphosphate (ATP) and reversibly form Cr-P. Cr and Cr-P form a creatine pool together, which plays a key role in the process of energy storage and utilization. As an energy transporter, Cr has higher mobility than ATP (12, 13). About 70 years ago, medical scientists managed to use GAA to treat human metabolic disorders and improve the working ability of manual workers, which directly proved that GAA has the effect in assisting the treatment of certain diseases (14).

**Figure 1:**
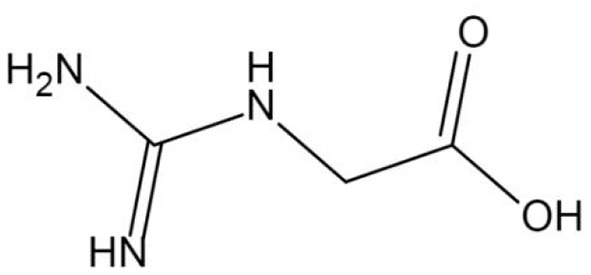
The structure formula of guanidinoacetic acid (GAA).

With the progress of research, the physico-chemical properties of GAA have been analyzed in detail. Industrial GAA usually appears as white or off-white crystalline powder without pungent odor. The chemical formula is C_3_H_7_N_3_O_2_ (relative molecular mass = 117.11 g/mol), and the structural formula is shown in Figure 1. When the sample temperature reaches 190°C, the chemical structure of GAA undergoes thermal decomposition, and the crystal melts as the temperature rises above 284°C. GAA has a high degree of stability in water, and the shelf life can be as long as 2 years, which also makes GAA more widely used than Cr (15).

The safety of GAA and Cr have been verified, and they have entered the public eyes as health care products and are favored by bodybuilders (16). Short or long-term intake of Cr will not harm kidney function or increase the risk of getting kidney disease, but exogenous intake of Cr supplementation is not recommended to somebody who has already been diagnosed with kidney disease (17). Other studies have also shown that Cr supplementation can weaken the excitatory of parasympathetic nerve of the heart, and the autonomic dysfunction may arise in even severe cases (18). However, in the process of livestock and poultry breeding, these substances are used to improve animal-growth performance and facilitate their muscle development and accumulation.

## Synthesis and Metabolism of Guanidinoacetic Acid

GAA is a precursor of Cr, an important compound in high-energy phosphate bioenergetics. Its synthesis in the body mainly occurs in the kidneys, it is also synthesized in other tissues such as the pancreas, liver, and muscle (19). The latest research hypothesized that the bacterial flora in the healthy gut can accelerate the synthesis of GAA by secreting enzyme guanidinoacetase (synthesis and hydrolysis occur simultaneously, synthesis > hydrolysis). If this hypothesis is verified, then there's a valid connection between the synthesis of GAA and microorganisms biological functions (20).

Generally speaking, the synthesis of GAA requires the presence of glycine and L-arginine. Under the catalysis of L-arginine:glycine amidinotransferase (AGAT), the two undergo amidino transfer to produce L-ornithine and GAA. GAA travels to the liver by the blood circulation through the portal vein, which sets the basis for the further formation of Cr. The source of GAA in the body comes from endogenous synthesis and food supplementation (negligible, 10 mg/kg of meat), while the consumption of GAA is caused by the synthesis of Cr and excretion by urine. The aim is to keep the GAA content stable at 2.6 ± 0.8 umol/L, which also constituted a theoretical model of GAA homeostasis (21). In the next step, GAA and S-adenosyl-methionine (SAM) catalyzed by guanidinoacetate N-methyltransferase (GAMT), in which GAA combined with a methyl group to generate Cr, and released S-adenosyl-homocysteine (SAH) at meantime. This process mainly occurred in the liver, but also in the skeletal muscle, spleen, brain and genital organs (22). Finally, Cr is released into the blood circulation and entered the cell through a specific transporter SLC6A8 (a Na^+^/Cl^−^ creatine co-transporter) located on the cell membrane (23). Since Cr can regulate the expression of AGAT through a counter-regulatory mechanism but cannot counter-regulate the expression of GAMT, the synthesis order of GAA and Cr cannot be reversed (24). In cells, Cr and ATP undergo a reversible reaction with the catalysis of creatine kinase (CK) to generate Cr-P and adenosine diphosphate (ADP) for energy storage (25). The body continues to metabolize Cr and Cr-p, and about 1.7% of them are metabolized into the final product—creatinine, which is transported from the blood to the kidneys and is completely filtered by the glomerulus, and is excreted in the urine. It is an important indicator of kidney function testing and is also commonly used to diagnosis of certain kidney diseases (26). The SAH produced during Cr production can be reversibly hydrolyzed to homocysteine and adenosine. Homocysteine is further decomposed into cysteine, which undergo the methylation reaction with its own methyl donors such as vitamin B. The methionine was formed by methylation, and then participates in the synthesis of Cr again (19).

**Figure 2:**
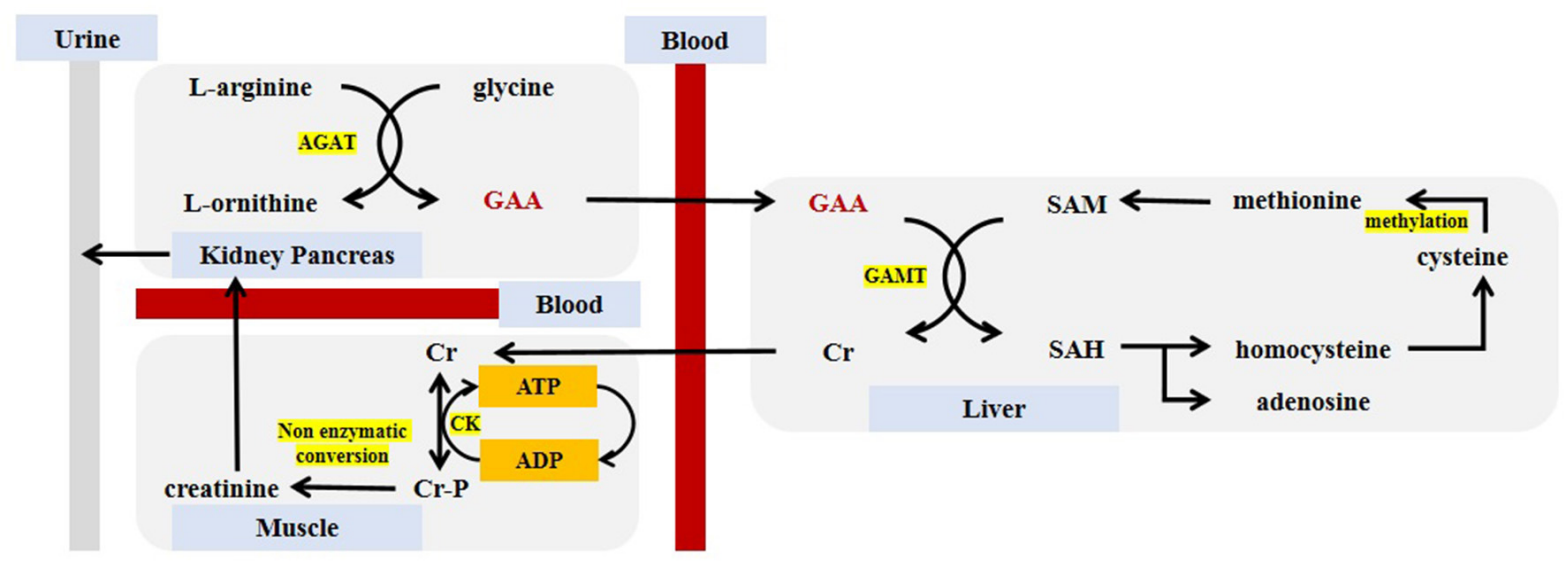
Guanidinoacetic acid synthesis and metabolism. GAA, guanidinoacetic acid; Cr, creatine; Cr-P, phosphocreatine; SAM, S-adenosyl-methionine; SAH, S-adenosyl-homocysteine; AGAT, L-arginine:glycine amidinotransferase; GAMT, guanidinoacetate N-methyltransferase; CK, creatine kinase; ATP, adenosine triphosphate; ADP, adenosine diphosphate.

Figure 2 describes the synthesis and metabolism of GAA in detail.

## The Regulation of Guanidinoacetic Acid on Skeletal Muscle Development

### Skeletal Muscle Classification and Structure

Taking the difference in biological function and structural composition as the classification basis, muscle tissue can be divided into two types: striated muscle and smooth muscle (27). Smooth muscle is mainly distributed in blood vessel walls, respiratory tract, digestive tract and other internal organs. It is an uncontrollable type of muscle regulated by autonomic nerves (28). The striated muscle is composed of skeletal muscle and myocardium. It is named because of the light and dark stripes that can be observed under the microscope, and the striated muscle are dually innervated by consciousness and the nervous system (29).

Skeletal muscles are also divided into fast muscles and slow muscles, and this classification is not limited to vertebrates. These two types of skeletal muscles are also found in invertebrates such as octopuses and crabs (30). Myofibers are cell units that make up muscle tissues and gather into hundles, while multiple muscle bundles gather to form muscles. In addition, the composition of myofibrils includes thin actin filaments and thick myosin filaments which are composed of specific proteins (responsible for muscle contraction) and arranged regularly in the sarcoplasm (3). Based on the metabolic capacity and contractility of myofibers, it can be summarized as aerobic metabolism myofibers (type I myofibers) and glycolytic metabolism myofibers (type II myofibers, including IIa, IIb, IIx). Type I myofibers are rich in myoglobin and cytochromes showing a brighter red color but have a slow contraction speed, while type II myofibers contract fast and appear white, among all types of type II myofibers, the type IIb has the fastest contraction speed (31).

### Skeletal Muscle Development

A large number of studies have shown that the development of skeletal muscle includes the following four processes: the differentiation of amniotic mesoderm stem cells (32) to generate myoblasts, the differentiation and fusion of myoblasts to generate myotubes, the formation of myofibers, and the maturation of myofibers (33). The number of myofibers in humans and animals has been determined before birth, but the expansion of their volume and the transformation of the types of myofiber depends on acquired comprehensive factors (34). In the process of skeletal muscle formation, the primary and secondary myotubes occur at different developmental stages (35). Primary myotubes are formed during the embryonic period and the number is closely related to genetic factors. The secondary myotubes begin to develop in the fetal period, and the nutritional regulation during maternal pregnancy can significantly affect the development of muscle fibers (36–38). In addition, since the secondary myotube grows around the primary myotube, the larger the diameter of the primary myotube, the more secondary myotubes can grow around it (39). Some research have shown that the development of primary myotubes eventually generated slow-twitch fibers, but the secondary myotubes generated fast-twitch fibers (40).

After the fetus is born, the myofibers gradually mature. At this time, muscle growth only depends on the changes in the volume of muscle fiber and the transformation between different types of muscle fiber (41). The reason for the increase in muscle fiber volume is related to the proliferation and differentiation of muscle satellite cells, which are muscle-derived stem cells that are normally in a resting state but have differentiation potential (42). The reason for the increase in muscle fiber volume is related to the proliferation and differentiation of muscle satellite cells, which are muscle-derived stem cells that are normally in a resting state but have differentiation potential (42). The muscle satellite cells exist widely between the muscle cell membrane and the matrix membrane, and have the biological function of promoting myomuscle regeneration after being activated (43, 44). Moreover, myofiber are a type of multinucleated cells (nuclei can reach several hundred), and each nucleus only controls certain part of the cytoplasm, referred to as myonucleus area (each cell nucleus and controlled cytoplasm are called a DNA unit). Satellite cells also have the ability to increase the number of myofiber nuclei to maintain the balance of the ratio between nucleus and cytoplasm (45).

### Guanidinoacetic Acid Regulates Genes Involved in Skeletal Muscle Development

In eukaryotes, differences in gene expression are the root cause of differences between individuals, gene expression is controlled at different stages of individual development and is affected by many factors (46). There are many genes that regulate the development of skeletal muscle, among which the myogenic regulatory factors (MRFs) plays an important role in the growth of skeletal muscle. It mainly includes four specific transcription factors, myogenic determining factor (MyoD), myogenic factor-5 (Myf5), myogenin (Myog), and muscle regulatory factor-4 (MRF4 or Myf6). The expression of MyoD and Myf5 contributed to the directed differentiation of myogenic cells, while Myog and MRF4 performed their functions in the differentiation process of myoblasts. It shows that different genes are expressed in the sequence of time during muscle development (47, 48). There are few molecular studies of GAA on muscle growth. We can explain the effect of GAA on muscle growth from its metabolite Cr.

CR supplementation can improve the expression of MRFs, especially in young individuals, but this effect gradually decreases with the increase of age (49). After treating C2C12 myoblasts with Cr, it was found that although the degree of influences on the expression of MyoD, Myf5, Myog, and MRF4 genes were different, they were all positive (50). Moreover, some studies have shown that Myf5, MyoD, or MRF4 inactivation can produce viable mice, but the absence of Myog causes the mice to die after birth (51).

Pax gene family is involved in all stages of muscle cell growth and differentiation, mainly including Pax3 and Pax7 (52). Pax gene family is involved in various stages of muscle cell growth and differentiation, including Pax3 and Pax7. The main functions of Pax include regulating the behavior of myogenic progenitor cells and the formation of skeletal muscle. Pax3 plays a dominant role in the above processes. The lack of Pax3 has caused the early embryonic development to be restricted, and impaired muscle regeneration in the later period (53, 54). And Pax3 can directly regulate the expression of MyoD and Myf5, and indirectly act in the differentiation process of myogenic cells (55, 56). In contrast, Pax7-missing led to slower muscle development, reduction of the amount of muscle tissue, whereas there is no pathological change in the structure (57).

Myostatin (MSTN) is an important member of transforming growth factor superfamily, also known as growth differentiation factor-8 (GDF-8), which is mainly manifested in the negative regulation of muscle growth and strength increase (58). MSTN can activate receptor function by binding to ALK4/5 and ActR2A/B type receptors on the surface of muscle cells, and lead to the function of promoting protein degradation and inhibiting protein synthesis in muscle (59). Specifically, the mature MSTN fragment first binds to type II receptor (mainly ActRIIB) and starts the signal cascade in muscle cells, which makes ActRIIB autophosphorylate and bind to type I receptor (ALK4 and ALK5) with low affinity to enhance the transcription process of target gene (60). In addition, MSTN can also inhibit the expression of protein kinase B (AKT) and the transcriptional activity of MyoD, and may lead to muscle atrophy and other diseases (61). However, the missing of MSTN or gene homozygous mutation result in abnormal accumulation of muscle mass and proliferation of myofibers (62). In the research field of skeletal muscle growth and development, MSTN is recognized as a negative regulator with important physiological functions (63). GAA and its metabolite Cr have the effect of down-regulating the expression of MSTN and eliminating its inhibitory effect on muscle growth (64).

Myocyte enhancer factor 2 (MEF2) gene family is another gene family in the body that can directly regulate skeletal muscle development in addition to the MRFs gene family. It is composed of four genes (65), MEF2a, MEF2b, MEF2c, and MEF2d, and is highly expressed in myoblasts. It functions mainly by recognizing a conserved A/T-rich elements in genes (66). MEF2c and Myog co-stimulate MyoD expression to activate the differentiation process of myoblasts (67, 68). Exogenous Cr supplementation helps the expression of MEF2 to improve muscle growth (69).

In addition, some scholars have found that peroxisome proliferator-activated receptor gamma coactivator 1α (PGC-1α) can increase the accumulation of skeletal muscle during exercise and regulate the transcription of some target genes (70).

MicroRNA (miRNA) is a very conservative non-protein coding RNA that can directly degrade target gene mRNA or inhibit its translation. It plays a key role as a post-transcriptional regulator in myogenesis (71). GAA induced the activation of AKT/mTOR/S6K signaling pathway through miR-133a-3p and miR-1a-3p to promote myoblast differentiation (72).

**Figure 3:**
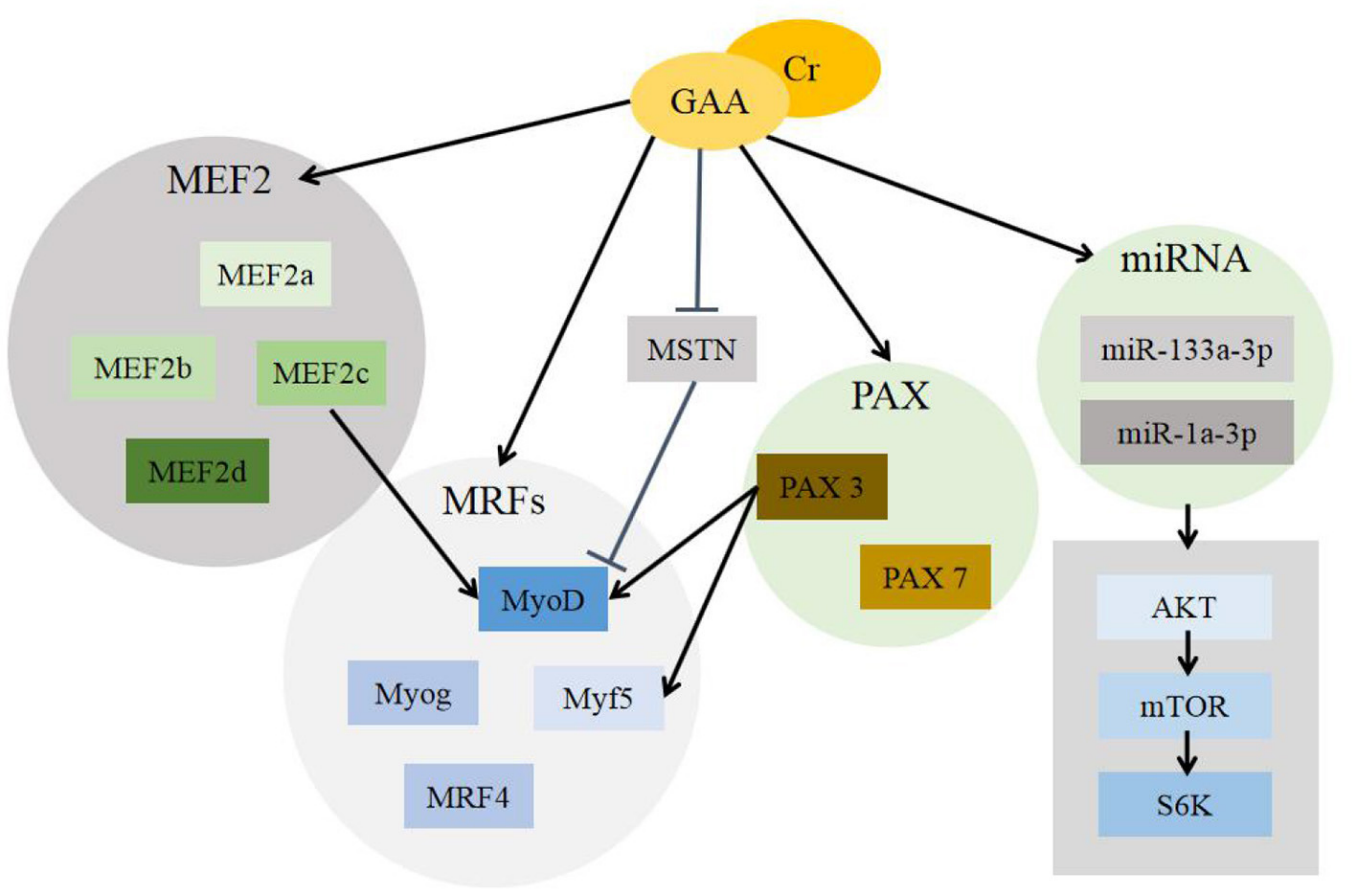
Mechanisms of GAA/Cr promoting skeletal muscle development and growth. GAA, guanidinoacetic acid; Cr, creatine; MEF2, myocyte enhancer factor 2 (includes MEF2a, MEF2b, MEF2c and MEF2d); MRFs, myogenic regulatory factors (includes MyoD, Myog, Myf5, MRF4); MSTN, Myostatin; PAX, Pax gene family (includes Pax3 and Pax7); miRNA, MicroRNA (includes miR-133a-3p and miR-1a-3p); AKT, protein kinase B; mTOR, mammalian target of rapamycin; S6K, S6 kinase.

Figure 3 describes the pathways regulated by GAA/Cr during their skeletal muscle development and growth.

## Application of Guanidinoacetic Acid in Animal Models

Since researchers recognized the importance of Cr for muscle development, many studies have been carried out to evaluate the effects of different amino acids and other substances on endogenous Cr metabolism. Researches added 1 g of GAA to the diet of the young rats in 1930s, and they found that the Cr level was increased by nearly 50% which reached the peak after 17–24 h. This effect was even higher than adding the equivalent amount of Cr (73). Vivo experiments proved that when the mouse body lacks Cr, GAA can provide energy for the body under the catalysis of CK. It also shows that CK can use GAA and go through the phosphorylation pathway to fight against energy damage (24, 74). And the main reason why Cr contributes to the synthesis of muscle tissue protein, improves muscle energy reserves and muscle strength is that it's synthesized with the precursor substance GAA. As a supplement to the aforementioned regulation of genes related to skeletal muscle development, GAA and Cr also functions by promoting the secretion of insulin-like growth factor-1 (IGF-1) and growth hormone (GH) in the body (both IGF-1 and GH are anabolic hormones that can increase muscle growth) (64). Vitro experiments have shown that GAA can promote the expression of MyoD and MyoG mRNA and increase the fusion rate of myotubes in C2C12 myoblasts. It can also affect the level of total myosin heavy chain (MyHC) protein to increase myotube thickness and gastrocnemius cross-section area (72). Another *in vitro* experiment demonstrated that Cr can activate the differentiation process of C2C12 myoblasts by activating the P38 and Akt/PKB cell pathways, which is manifested by the expression of myosin heavy chain type II, the increase in the number of nuclei in the myotube, and promote the occurrence of the cell fusion process (75).

Returning to the perspective of animal husbandry and nutrition. With the development of the global economy and the continuous improvement of consumption levels, human being to pay more and more attention to the nutritional balance of daily diet (76). Due to the demand for high-quality protein, the demand for high-quality meat is also increasing. Therefore, enhancing the amount and speed of muscle accumulation in livestock and poultry is the most crucial section in the development of animal husbandry (77).

In addition to affecting the growth of muscles, the content of Cr in the animal body can also maintain the steady state of ATP and buffer the accumulation of lactic acid in the muscles (78), and improve meat quality. The methods for animals to obtain Cr can be categorized as endogenous and exogenous. The endogenous method mainly generates GAA through arginine and glycine, and then synthesizes Cr with methionine in the liver, but this does not meet the animals' needs (79). Exogenous Cr sources mainly include animal protein raw materials (meat and bone meal) and fish protein raw materials (fish meal) (80), while plant raw materials lack Cr or its precursor. Combined with the analysis of animal feed composition and nutritional level, it can be found that animals cannot get enough Cr. So they can only synthesize Cr at the cost of consuming endogenous amino acids, resulting in the loss of amino acids (81). Especially taking the application of methionine in poultry production as an example, methionine is the first limiting amino acid of poultry and is very beneficial to the growth of muscles and feathers, and the consumption of methionine in the process of Cr synthesis has resulted in increased demand for methionine in poultry (82). Moreover, for young animals, arginine is an essential amino acid with growth promoting effect. Lack of arginine may easily cause the slow growth of chicks, while adding GAA to arginine-deficient diets can significantly improve the growth performance of chicks. It is suggested that GAA can be a good substitute for dietary arginine (83).

Using Cr as feed additive directly has problems of poor stability, high cost and low animal bioavailability. The use of GAA will solve these problems (84). In addition, animals have a very high utilization efficiency of exogenous GAA. After experiments with colon-fistulated broilers, it was found that the digestibility-rate of GAA reached 99% (85). GAA and its metabolite Cr not only improve animal growth performance and promote muscle growth, but also affect meat quality (10). It shows that the drip loss of meat is reduced, the yellowness is increased, and the activity of the free radical metabolism related enzymes and the antioxidant enzymes (reducing lipid peroxidation) are improved (86).

Table 1 summarizes the results of using GAA or Cr in livestock production.

**Table 1:** The results of using GAA or Cr in livestock production.

**Experiment material**	**Compound**	**Observed effects**	**References**
Duroc × Landrace × Yorkshire pigs	GAA	ADG↑, ADFI↑, lean meat percentage↑, back fat thickness↓, MYH4↑, MyoD↑, Myf5↑, MSTN↓.	(87)
Yucatan miniature pigs	GAA or Cr	Liver: both can cause the Cr concentration↑. Muscle tissue: GAA can cause the Cr concentration↑.	(82)
Duroc × Landrace × Yorkshire pigs	GAA	Muscle tissue: Cr↑, ATP↑, carcass weight and lean meat percentage↑.	(88)
Finishing pigs	GAA or CMH	Both can cause the Cr↑, Cr-p↑, creatine transporter mRNA↑, myofibrillar protein solubility↑.	(89)
Duroc × Landrace × Large White male pigs	GAA or GAA+betaine	Meat quality↑, Cr↑, Cr-P↑, ATP↑, CK↑, creatine transporter mRNA↑.	(90)
Finishing pigs	CMH	Drip loss↓, meat color L*↑.	(91)
Male Ross 708 chicks	GAA	Serum Cr↑, muscle Cr-P↑, glycogen↑, growth performance↑, muscle energy stores↑.	(92)
Male Ross 308 chicks	GAA	Weight gain↑, FCR↑, Heart and breast muscle ATP/AMP ratio↑.	(93)
Broiler	GAA	Live weight↑, breast meat percentage↑, meat quality↑.	(94)
Arbor Acres broiler	CrPyr	Live weight↑, breast meat weigh↑t, Cr↑, Cr-P↑, CK↑.	(95)
Balady chicks	CMH or CMH+Znic	Live weight, weekly bodyweight gain, feed efficiency, carcass weight. CMH+Znic are better than CMH.	(96)
Mulard ducks	GAA or GAA+Met	Weight gain↑, IGF-1↑, GH↑, Myog↑, MSTN↓, GAA+Met better than GAA only.	(97)

ADG, the average daily gain; ADFI, the average daily feed intake; FCR, feed conversion ratio; GAA, guanidinoacetic acid; Cr, creatine; Cr-P, phosphocreatine; CMH, creatine monohydrate; CrPyr, creatine pyruvate; Met, methionine; ATP, adenosine triphosphate; CK, creatine kinase; IGF-1, insulin-like growth factor-1; GH, growth hormone; MYH4, myosin heavy chain gene; MyoD, myogenic determining factor; Myog, myogenin; MSTN, Myostatin. ↑up-regulation, ↓down-regulation, L*, lightness.

Apart from synthesizing Cr to promote protein deposition and muscle growth in the body, the apply of GAA also has the effect of promoting insulin secretion to control blood glucose (98). Insulin is the only hormone in the body that plays a role in lowering blood glucose, and it is also the only hormone that contributes to glycogen, protein and fat synthesis. It links the regulatory effect of GAA intake on insulin with muscle growth (99, 100). The safety of GAA as a dietary supplement was evaluated in the initial human studies, and subsequent clinical trials have also proved that GAA is a non-toxic and highly tolerable substance. Dietary GAA supplementation in humans leads to an increase in serum creatinine levels without impairment of renal function (15). Some studies also shown that a small number of samples that take GAA will have an enhancement of serum homocysteine. This might be a potential adverse effect, since hyperhomocysteinemia is considered as an independent risk factor for cardiovascular and atherosclerotic diseases (101). Exogenous intake of GAA mainly exhibits antioxidant effects at low doses, while pro-oxidative effects and even oxidative stress appear at high doses (102). GAA is a kind of pro-oxidant (103), but its metabolites such as Cr and arginine all express anti-oxidant effects, so GAA is also regarded as an indirect antioxidant (104). However, the relationship between the level of GAA intake and the pro-oxidant-antioxidant properties needs to be further elucidated.

## Perspectives

A large number of animal experiments and human clinical applications have proved that GAA has good effects in improving muscle development and growth. Especially in animal husbandry, it has been widely used, and has the advantages of low cost relative to Cr and arginine, and better growth promoting effect. However, the research on GAA are not very in-depth, directions such as its mechanism of promoting muscle development, the determination of the effective dosage in animal production, and whether GAA has toxic effects or not have not been fully studied. Therefore, more detailed research work are needed.

## Author Contributions

ZhaomY, ZhaoyY, and SL are the primary investigator in this study. YY and TY are participated in literature collection and summary. ZhaoyY and QC revised the manuscript. QC designed this study and wrote the manuscript. All authors have read and approved the final manuscript.

## Conflict of Interest

The authors declare that the research was conducted in the absence of any commercial or financial relationships that could be construed as a potential conflict of interest.

## Publisher's Note

All claims expressed in this article are solely those of the authors and do not necessarily represent those of their affiliated organizations, or those of the publisher, the editors and the reviewers. Any product that may be evaluated in this article, or claim that may be made by its manufacturer, is not guaranteed or endorsed by the publisher.
